# Bottom-Up Effects of Drought-Stressed Cotton Plants on Performance and Feeding Behavior of *Aphis gossypii*

**DOI:** 10.3390/plants12152886

**Published:** 2023-08-07

**Authors:** Jinping Liu, Chen Wang, Huatong Li, Yu Gao, Yizhong Yang, Yanhui Lu

**Affiliations:** 1 State Key Laboratory for Biology of Plant Diseases and Insect Pests, Institute of Plant Protection, Chinese Academy of Agricultural Sciences, Beijing 100193, China; 13720142405@163.com (J.L.); chenwang_wc@163.com (C.W.); lihuatong163@163.com (H.L.); gaoyu0418@126.com (Y.G.); 2 College of Plant Protection, Yangzhou University, Yangzhou 225007, China

**Keywords:** climate change, drought, plant–insect interactions, detoxification enzyme, feeding behavior, life history

## Abstract

Drought, a major stress for crop plants, is expected to increase in frequency due to climate change. Drought can alter crop growth and levels of secondary plant metabolites, which in turn can affect herbivores, but this latter point is still controversial. This study used three different polyethylene glycol (PEG-6000) levels (0%, 1%, and 3%) to simulate drought stress and evaluated their effects on cotton plants and the impacts on the performance of the cotton aphid *Aphis gossypii*. Cotton plants under drought stress showed decreased water content, above-ground biomass, and nitrogen content and increased soluble protein, soluble sugar, and tannin contents. Based on analysis of the developmental time and fecundity data from individuals and at the population level, a significantly lower fecundity and population abundance of *A. gossypii* were detected on cotton plants with drought stress, which supports the “plant vigor hypothesis”. The poor development of *A. gossypii* is possibly related to lower xylem sap and phloem ingestion under drought stress. In addition, the increased tannin content of cotton plants induced by drought and lower detoxification enzyme activities of *A. gossypii* may have affected the responses of aphids to drought-stressed plants. Overall, the results showed that drought stress altered the physiological characteristics of the cotton plants, resulting in adverse bottom-up effects on cotton aphid performances. This implies that the adoption of drip irrigation under plastic film that can help alleviate drought stress may favor the population growth of cotton aphids.

## 1. Introduction

Drought is a worldwide agricultural problem that can adversely affect plant growth and crop yield that has caused global losses in crop production totaling ~30 billion USD in the past decade [1]. The frequency and intensity of droughts are predicted to increase in the future, mainly due to climate change [2]. This predicted change is expected to drive shifts in the physiological, physical, and chemical characteristics of plants, which in turn will affect the population numbers and growth of insect herbivores [3].

Through long-term evolutionary processes, plants have developed adaptative mechanisms to cope with unfavorable conditions like drought at the cellular, physiological, biochemical, and molecular levels [4,5]. At the cellular level, when plants are subject to water deficit, cellular water loss is the first event to occur [6]. Drought stress usually reduces leaf water content and water potential in leaf tissues, which can reduce the turgor pressure of cells [7]. As previously reported, phloem-feeding insects require plant cells with a high turgor pressure to extract adequate nutrients [8,9]. Thus, the lower water status of leaves during drought may negatively affect the ability of such insects to obtain or use nutrients [5]. During drought stress, the accumulation of osmosis-regulating substances in plants, such as soluble proteins and soluble sugars, is regarded as a common physiological response, which could mitigate the damage from drought stress by sustaining turgor and metabolism activities in cells. This same process may also alter the nutritional quality of insects [10,11]. Additionally, increased accumulation of C-based secondary metabolites (e.g., tannins and phenolics) in plants was found to occur in response to drought conditions [12,13], which might also deter attacks of insect herbivores [14]. Furthermore, drought stress frequently increased the N content of plants, which has been demonstrated in many studies to favor population growth of phloem-feeding insects [15]. Thus, drought may affect plant nutritional quality, palatability, and levels of defensive compounds against insect herbivores. To date, there are three widely recognized hypotheses to explain the relationship between insect performance, plant quality, and drought. First, the plant stress hypothesis [15] argues that insect herbivores' abundance is higher in drought-stressed plants because of an increase in the availability of nutrients and a reduction in the concentrations of defensive compounds. However, the plant vigor hypothesis [16] predicts better performance of insect herbivores on vigorous plants than on drought-stressed plants. Third, the pulsed stress hypothesis [6] predicts that pulsed or intermittently drought-stressed plants are beneficial to the performance of phloem-sucking feeders due to an increase in the availability of nitrogen but also the periodic recovery of turgor pressure compared to continuously stressed plants. Thus, there are competing views on how drought stress influences the plant–insect interaction.

Because aphids are phloem feeders, they are considered to be more susceptible to water changes in their host plants. Thus, an increased frequency or intensity of drought events in future climates could affect the population growth and development of aphids. Past studies suggest that aphids’ responses to drought stress are “species-specific” and can be negative, positive, or neutral depending on the host plant, insect guild, and drought intensity [17]. For example, drought stress promoted the population growth of the generalist aphid *Myzus persicae* when reared on *Arabidopsis thaliana* plants but did not significantly affect the population growth of the specialist aphid *Brevicoryne brassicae* [18]. Simpson et al. [19] found that the population numbers of *M. persicae* reared on *Brassica oleraceae* var. *capitata* were negatively associated with drought intensity. However, Tariq et al. [20] found that medium levels of drought stress on *B. oleraceae* var. *capitata* were beneficial to the population growth of both *M. persicae* and *B. brassicae*. Recently, a meta-analysis by Leybourne et al. [17] found that drought stress had negative impacts on aphid fitness, which were associated with reduced plant vigor (e.g., lower biomass and growth rate) and increased chemical defenses in drought-stressed plants. 

The cotton aphid, *Aphis gossypii*, is a polyphagous insect infesting nearly 100 crop species throughout the world [21] that causes extensive agricultural crop damage through direct feeding and transmission of plant viruses [22,23]. This aphid is the predominant pest in the cotton regions of China, especially in Xinjiang province [24]. Drought stress is one of the major abiotic stresses limiting cotton growth and production, and drought severity has been increasing in the cotton regions of China since the 1980s, reducing cotton yield [25]. Moreover, before the 1990s, the irrigation of cotton in the Xinjiang cotton region was primarily through flood irrigation. After flood irrigation, the soil water evaporates quickly and soil water content is extremely variable, resulting in frequent intermittent drought stress on cotton plants, reducing cotton growth and yield. Since its introduction and widespread adoption, drip irrigation under plastic film has effectively eliminated such intermittent drought events. With the new technology, the soil water level remains relatively stable and provides sufficient water for cotton growth. Given the plant vigor hypothesis, whether these changes to cotton irrigation have promoted the outbreaks of *A. gossypii* seen in recent decades is still unclear. For these reasons, clarifying the responses of *A. gossypii* to drought-stressed cotton is an important objective for reducing aphid damage to cotton. Although many studies have focused on the responses of cotton plants to drought stress (e.g., Parimala and Muthuchelian, 2010; Megha et al., 2017; Zou et al., 2020) [25,26,27], a few have examined how *A. gossypii* responds to variations in drought stress in cotton plants. Sconiers and Eubanks [28] found that *A. gossypii* abundance decreased when it was reared on cotton grown under severe drought stress based on two years of field trials, and furthermore, the negative effects of drought on *A. gossypii* abundance were positively associated with changes in the amino acid concentrations in cotton leaves. However, the responses of life history, feeding behavior, and the physiological metabolism of *A. gossypii* to changes in cotton treated with drought stress induction are still unknown.

Polyethylene glycol 6000 (PEG-6000), an inert and non-ionic macromolecular compound with a high impermeability, has been widely employed as an osmotic regulator to induce water stress and simulate water potential. Extensive studies have demonstrated the efficacy of PEG-6000 in investigating the impact of drought stress on diverse crop species, such as cotton, rice, corn, and others [29,30,31]. Here, three concentrations of PEG-6000 were utilized to simulate drought stress. We determined the direct effects of drought stress on cotton plants and the subsequent bottom-up effects on *A. gossypii*, as well as the potential physiological mechanisms driving the cotton plant–aphid interactions, using controlled tests in the laboratory. Our study goals were to (1) explore the changes in leaf water content, plant biomass, and physiology in cotton under drought stress; (2) evaluate the population fitness of *A. gossypii* reared on cotton plants under drought stress for both individuals and populations; (3) characterize the feeding behavior of *A. gossypii* on drought-stressed cotton plants; and (4) determine the activity levels of detoxification enzymes in *A. gossypii* fed on drought-stressed plants.

## 2. Results

### 2.1. Relative Water Content of Leaves and Above-Ground Biomass of Cotton Plants

**Figure 1:**
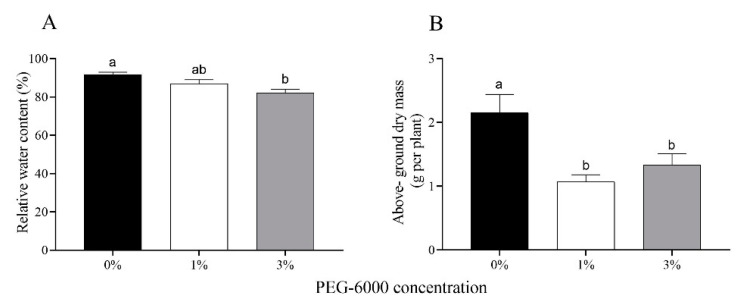
Relative water content of leaves (**A**) and above-ground dry mass (**B**) of uninfested cotton plants after 28 days of treatments with different PEG-6000 concentrations. Values are means ± SE of three replicates. Different small letters represent statistical differences between treatments (Tukey’s multiple range test, *p* < 0.05).

The relative water content of cotton leaves gradually decreased with increasing drought stress; specifically, it was reduced by 4.8% and 9.6% for the 1% and 3% PEG-6000 treatments (Figure 1A). In response to drought stress, the above-ground biomass of cotton plants treated with 1% and 3% PEG-6000 stress was reduced 2.00- and 1.62-fold compared to the untreated control (Figure 1B).

### 2.2. Soluble Sugar, Soluble Protein, Nitrogen, and Tannin

**Figure 2:**
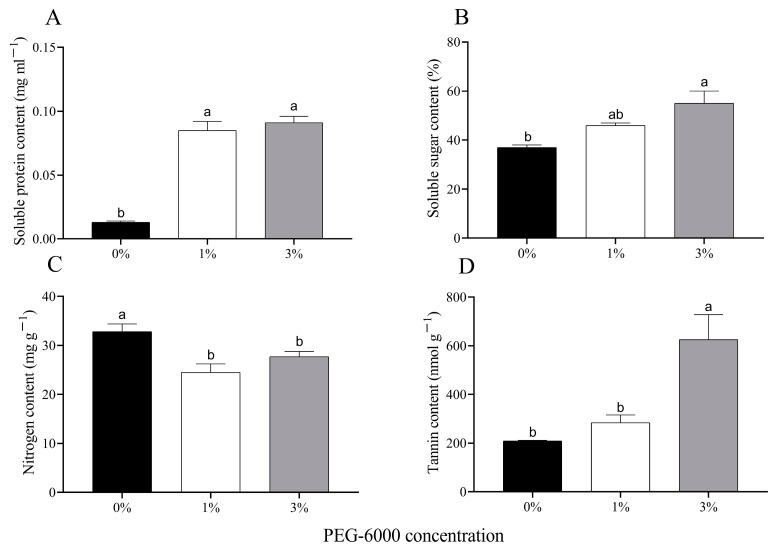
Changes in chemical composition of uninfested cotton plants treated with different PEG-6000 concentrations. Values are means ± SE of three replicates. (**A**) Soluble protein content. (**B**) Soluble sugar content. (**C**) Nitrogen content. (**D**) Tannin content. Different small letters represent statistical differences between treatments (Tukey’s multiple range test, *p* < 0.05).

The soluble protein content of cotton leaves increased significantly under water stress when plants were grown in 1% or 3% PEG-6000 drought treatments (*F* = 79.090, *df* = 2, 6, *p* < 0.0001) (Figure 2A). The soluble sugar content of cotton leaves grown in the 1% and 3% PEG-6000 drought-stress treatments increased by 24.3% and 48.6%, respectively (Figure 2B). The nitrogen contents of cotton leaves grown in both of the two drought-stress treatments were significantly reduced compared to the control (Figure 2C). The 3% PEG-6000 drought-stress treatment significantly increased the tannin content of the cotton leaves, but the 1% PEG-6000 drought-stress treatment did not do so.

### 2.3. Aphid Life History Parameters

**Figure 3:**
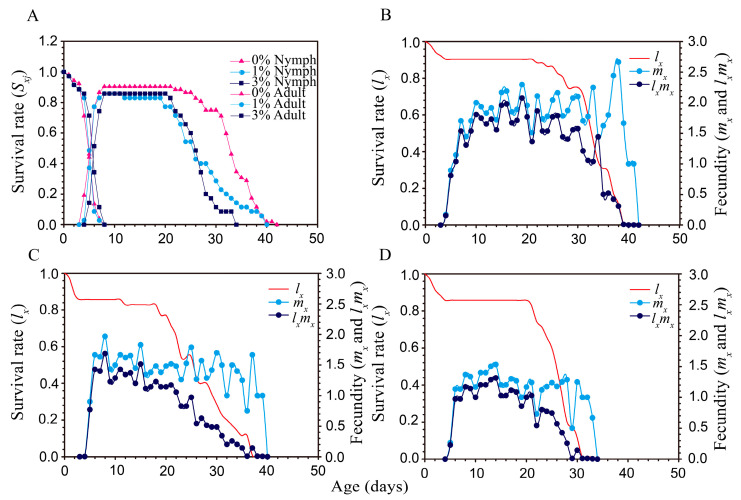
Survival rate and fecundity of *Aphis gossypii* under different PEG-6000 concentrations. (**A**) Age-stage specific survival rate *(s_xj_*); (**B**–**D**) were the agespecific survival rate (*l_x_*) and fecundity (live births) (*m_x_* and *l_x_m_x_*) of *A. gossypii* fed on cotton plants grown under 0%, 1%, and 3% PEG-6000 concentrations, respectively.

The effects of drought-stressed plants on the development and growth rates of individual cotton aphids (Table 1 and Table 2; and Figure 3) showed that the developmental time of the nymphal stage was significantly longer under the high-drought-stress treatment (3% PEG-6000) than either the control or the 1% PEG treatment (Table 1). Adult longevity was significantly shorter under both drought-stress treatments (1% and 3% PEG-6000) than the control. There was no significant difference in the adult pre-reproductive period (APOP) of the aphids reared on the 1% vs. 3% PEG treatments, but both of these drought treatments shortened the APOP to about one-third of that of the control (0% PEG-6000). Drought stress significantly affected the fecundity of cotton aphids. The number of offspring produced by adults reared on the high-drought treatment (3% PEG-6000) plants was about half of that of the control plants. The population growth rates (intrinsic rate of increase (*r*) and finite rate (*λ*)) of the aphid populations were lowest on the high-drought-stressed plants (3% PEG-6000) compared to the 1% PEG-6000 stress or the control (Table 2). Consequently, the *R*_0_ values of the aphid populations decreased significantly with increasing drought stress (*p* < 0.05).

The age stage survival rate (*S_xj_*) of aphids (Figure 3A) shows the survival rate, stage differentiation, and variable developmental time. Drought stress did not significantly affect the pre-adult survival rate of aphids, but after 8 d on drought-stressed plants (either 1% or 3% PEG-6000), the survival of adult females was lower than in the control treatment (Figure 3A). Differences in daily fecundity among the PEG-6000 stress were shown by the age-specific daily fecundities (*m_x_*) (Figure 3B–D). Peak *m_x_* values were 2.67 offspring/day (at an age of 38 days) in the control (Figure 3B), 1.97 (at 8 days) in the 1% PEG-6000 treatment (Figure 3C), and 1.37 (at 8 days) in the 3% PEG-6000 treatment (Figure 3D).

**Figure 4:**
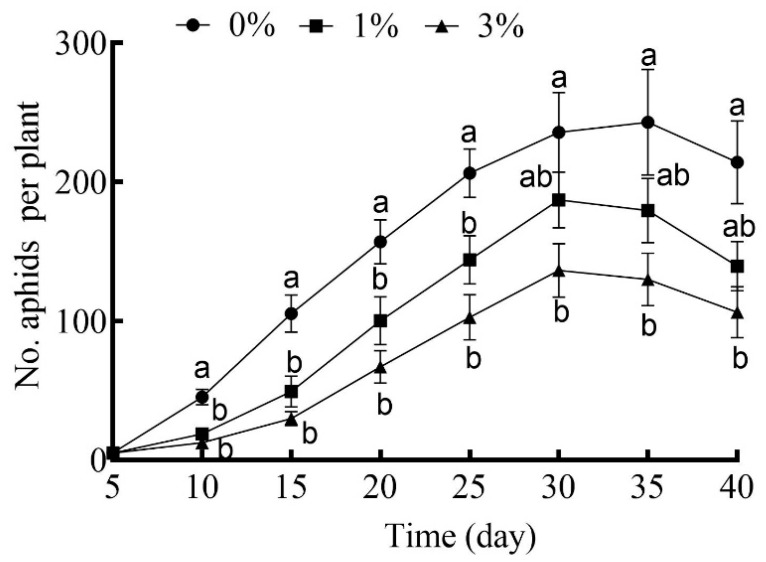
The number of cotton aphids in cohorts reared on cotton plants grown under different drought-stress levels with 0% PEG-6000 being the control and aphids censused every 5 days. Values are means ± SE of 15 replicates. Different small letters represent statistical differences between treatments (Tukey’s multiple range test, *p* < 0.05).

The effects of drought stress in general were to depress the growth rates of cotton aphid cohorts over time (Figure 4). The number of aphids in the cohorts reared on plants under the higher level of drought stress (3%) was consistently reduced on all census dates, but this was not the case for the lower level of drought stress (1%) (Figure 4).

### 2.4. Changes in Metabolic Enzyme Activity of Aphids

**Figure 5:**
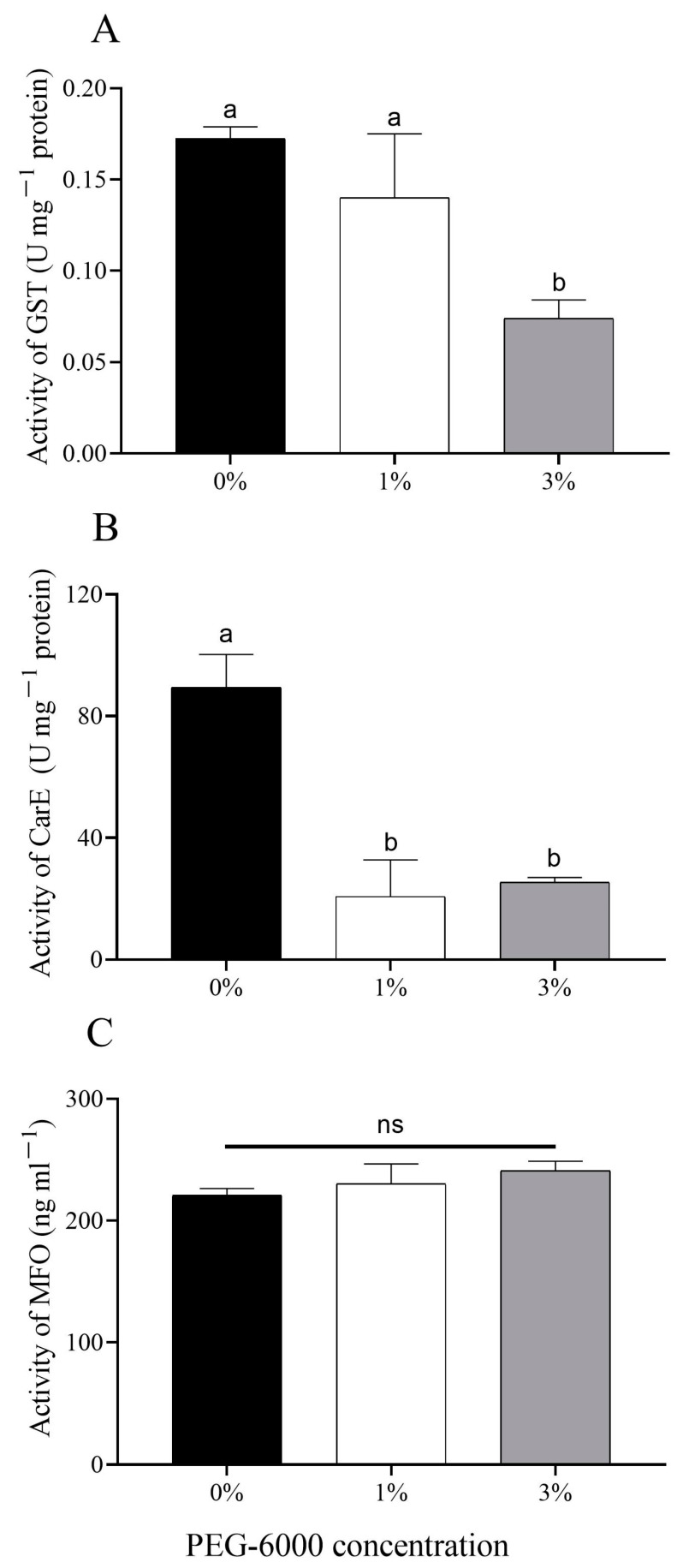
Changes in levels of metabolic enzymes of cotton aphids grown on cotton plants treated with different levels of PEG-6000 stress. (**A**) Glutathione-S-transferase (GST); (**B**) carboxylesterase (CarE); (**C**) mixed-functional oxidase (MFO). Values are means ± SE of three replicates. Different small letters represent statistical differences between treatments. “ns” represent no statistical differences between treatments (Tukey’s multiple range test, *p* < 0.05).

There was a significantly lower glutathione-S-transferase (GST) activity of aphids fed on cotton plants treated with 3% PEG-6000 (*F* = 5.602, *df* = 2, 6, *p* = 0.038) compared with 0% PEG-6000 (Figure 5A). The activity of carboxylesterase (CarE) of aphids under the two drought-stress treatments was significantly decreased (*F* = 16.777, *df* = 2, 6, 1%: *p* = 0.005; 3%: *p* = 0.007) when compared with the control group (Figure 5B). Drought-stress cotton plants had no effects on the mixed-functional oxidase (MFO) activity of aphids (*F* = 0.839, *df* = 2, 6, *p* = 0.477) (Figure 5C).

### 2.5. Aphid Feeding Behaviors

**Figure 6:**
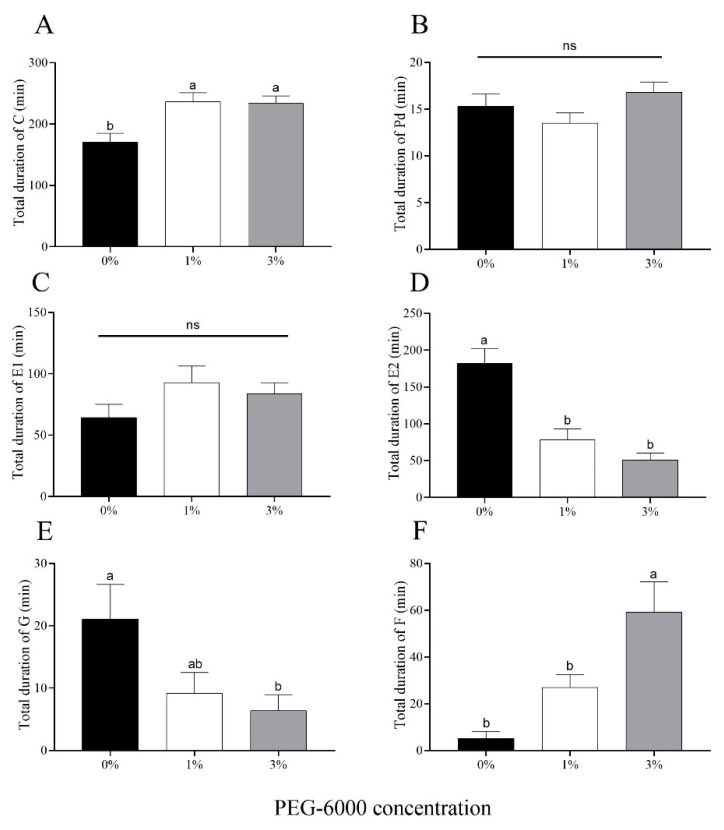
Electronic penetration graph (EPG) values for cotton aphid behaviors for aphids feeding on cotton plants grown under two levels of drought stress (1% and 3% PEG-6000 concentration) or the non-stressed control (0% PEG-6000 concentration). (**A**) Total duration of Behavior C (initiation of stylet penetration of leaf tissue). (**B**) Total duration of Behavior Pd (short bouts of intracellular penetration, which leads to a signal potential drop). (**C**) Total duration of Behavior E1 (secretion of saliva into phloem sieve elements at the beginning of the phloem phase). (**D**) Total duration of E2 (time spent phloem feeding). (**E**) Total duration of Behavior G (time spent xylem feeding). (**F**) Total duration of Behavior F (time lost to mechanical difficulty in stylet penetration). Values are means ± SE of 15 replicates. Different small letters represent statistical differences between treatments. “ns” represent no statistical differences between treatments (Tukey’s multiple range test, *p* < 0.05).

Drought stress significantly prolonged the average duration of Behavior C (initiation of stylet penetration of leaf tissue) for *A. gossypii* (Figure 6A). There were no significant differences in the total duration of Behavior Pd (short bouts of intracellular penetration, which leads to a signal potential drop) or Behavior E1 (secretion of saliva into phloem sieve elements at the beginning of the phloem phase) among the two water-stressed treatments or the control (Figure 6B,C). Cotton aphids fed on cotton plants grown on plants subjected to either of the drought-stress treatments (1% and 3% PEG-6000) showed a reduction in time spent phloem feeding (Behavior E2) (56.9% for the 1% treatment and 72.1% for the 3% treatment) compared to aphids reared on control plants not subjected to water stress (Figure 6D). For Behavior G (time of ingestion of xylem sap), the time for aphids in the control treatment was 2.29-fold and 3.30-fold greater than the times for the 1% and 3% PEG-6000 drought-stress treatments, respectively (Figure 6E). Furthermore, *A. gossypii* encountered more mechanical difficulty in stylet penetration (Behavior F) feeding on plants treated with 3% PEG-6000 stress compared to the other two treatments (Figure 6F).

## 3. Discussion

Drought occurs frequently in Xinjiang’s cotton region, which markedly affects the growth and metabolism of cotton plants. As previously reported, we also found that drought stress can reduce the relative water content of plant leaves [5,32]. In this study, we also showed that the drought-stress treatments tested significantly reduced the above-ground dry biomass, suggesting the growth of cotton plants was suppressed by water deficits, which is consistent with previous studies [25]. Here, we report that the population growth of *A. gossypii* cohorts was higher on vigorous cotton plants not subjected to drought stress, which supports the “plant vigor hypothesis”. These results agree with those of Sconiers and Eubanks [28] who found a lower performance of cotton aphids on drought-stressed host plants. These relationships suggest that drip irrigation under plastic films, which increases water retention by soil and promotes the growth of cotton plants, may be one of the causes of outbreaks of cotton aphids in regional cotton fields. However, Khederi et al. [33] found that the *A. gossypii* population was increased rather than decreased on drought-stressed pepper plants (*Capsicum annuum*), suggesting that drought may affect different aphid–plant interactions differently on different plants.

When plants suffer from water deficit, large amounts of osmosis-regulating substances accumulate in cells. This causes low osmotic potential, which allows external water to be absorbed to ensure normal metabolic activity. Soluble proteins and sugars are important osmosis-regulating substances in plants that increase under drought conditions [5]. We found that these groups increased during drought stress on cotton plants, and in principle, such compounds might also improve cotton’s nutrition quality to aphids but depend on the availability of aphids. From the perspective of the plant’s physiology, the decrease in above-ground dry biomass that we observed, together with the increase in soluble protein, indicates that drought-stressed cotton plants show lower rates of protein synthesis and assimilation into structural growth, possibly accompanied by synthesis of drought-induced proteins [10].

We found that the N content of the foliage of the drought-stressed cotton was lower than the control. Although the view that foliar N content increases in response to drought stress has been widely accepted, there are several other studies that have also found a decrease in the N content of drought-stressed plants [12,34,35]. Also, some studies have found no difference between drought-stress treatments and control plants [36,37]. This variation in outcomes may be caused by the nonlinear effects of drought stress on plant chemistry [35,38]. Previous studies have shown that any decrease in the N content of plant tissues reduced the plant quality for herbivorous insects, which are typically N-limited [39,40,41]. A meta-analysis by Huberty and Denno [6] showed that despite the fact that drought-stressed plants had elevated foliar N concentration, there was typically a strong negative effect on the performance of sap-feeding insects on drought-stressed plants. Thus, it is important to bear in mind that the N concentration in plants and an herbivore’s ability to access and utilize it are both important in determining a sap-feeder’s performance.

Significantly increased tannin levels were observed in drought-stressed cotton plants without aphid infestation treated with 3% PEG, indicating that tannin might participate in the cotton defense system against drought stress. Tannic acid is associated with resistance to *A. gossypii*, indicating that tannic acid exerts detrimental effects on *A. gossypii* [42]. In addition, the increased tannin content of cotton plants under drought stress may increase the phloem defense during aphid penetration [12]. Commonly, plant secondary defenses, such as elevated tannin levels, are induced by insect feeding [43]. For example, Wu et al. [44] found that the condensed tannin concentration was significantly elevated in cotton plants infested with *A. gossypii*. Here, we suspected that the tannin content was higher in drought-stressed cotton plants with aphid infestation compared to cotton plants without aphid infestation. Therefore, it may be that some of the adverse effects of drought on the life table parameters of *A. gossypii* are related to an increase in tannin in stressed cotton plants. In the long term, insect herbivores adapt to plant defensive compounds with increased detoxification abilities [43]. GST, CarE, and MFO are important insect detoxification enzymes. Although these enzymes have most frequently been linked to the ability of pests to detoxify pesticides, their role in detoxifying plant secondary metabolites has also been studied [44,45,46]. Previous studies have reported elevated levels of secondary plant metabolites such as phenols in drought-stressed cotton plants [13] and linked to reduced performance of *A. gossypii* [42]. Therefore, it is likely that the effects of drought stress in cotton would elevate levels of defensive metabolites and change the levels of detoxifying enzymes in herbivores such as *A. gossypii*. In the present study, significantly lower activity was observed for the enzymes GST and CarE in the *A. gossypii* fed on the cotton plants treated with 3% PEG-6000 compared to the control, suggesting that severe drought stress has negative effects on the detoxifying enzymes of *A. gossypii*. To sum up, the poor development of *A. gossypii* on drought-stressed plants may be attributed to the increased levels of tannins in the cotton plants and decreased levels of detoxifying enzymes in *A. gossypii*. In that context, any changes in cotton’s plant defensive metabolites under drought stress that also affects its resistance to aphids or other sap-sucking insects should be considered when breeding for enhanced drought resistance in cotton cultivars. Previous studies showed that aphid infestation may induce the chemical composition (terpenes) and nutritional alteration (such as sugar and amino acids) of phloem sap [47], which in turn may affect the responses of plants to drought stress. Thus, it can be seen that drought stress can impact the plant–aphid interaction that has the potential to change community structure. In the future, the plant–aphid interaction under drought needs more studies.

Aphids must ingest xylem sap to avoid dehydration and balance the osmotic pressure of the sugar-rich sap ingested during phloem-feeding because the osmotic pressure of phloem is higher than that of aphid hemolymph [48,49]. Thus, the occasional ingestion of xylem can increase the efficiency of aphid phloem feeding [50]. Nevertheless, aphid xylem feeding requires that plants have a relatively high water potential given that feeding is physically passive [51]. Thus, the lower xylem-feeding periods of *A. gossypii* on plants treated with 3% PEG-6000 may be due to the lower leaf water content, which in turn may have caused the lower aphid phloem ingestion (E2) observed. Phloem ingestion (E2) has been regarded as host–plant acceptance [12]. The shorter time in E2 of aphids fed on drought-stressed plants means the host plant was unsuitable. Phloem sap provides aphids with nutrients such as sugar, protein, and N, which are needed for growth and development [52]. In turn, the lower population abundance of *A. gossypii* fed on drought-stressed plants might have been due to a reduction in time spent in phloem-feeding (E2) and the consequence reduction in nutrition for growth. Thus, the host plant’s water status plays an essential role in the performance of aphid populations during drought stress. Behavior F occurs anywhere in the leaf tissue. In the current study, the mechanical difficulty in aphid stylet penetration (F) was significantly increased under drought conditions, which means that the leaf anatomical characteristics may have changed. Previous studies showed that drought changes the spongy mesophyll, epidermis, xylem number, and cell arrangement of plant leaves [53,54], but how the anatomical structure of the leaf affects aphid feeding remains to be further studied.

In conclusion, our study provides details on the effects of continuous drought stress on the physiology and chemistry of cotton and how those host plant changes affect the development, growth rate, and feeding behavior of *A. gossypii*. These results show that the growth of cotton plants was suppressed by the water deficit and that *A. gossypii* developed poorly on the stressed cotton, which supports the plant vigor hypothesis. Therefore, we argue that the adoption of drip irrigation under the plastic film or other farm operations in Xinjiang cotton fields that can help alleviate drought stress may favor increased growth rates of cotton aphid populations to some extent. However, even though the nutrient content, like the soluble sugar and protein levels, were improved in the drought-stressed cotton, we observed the cotton aphids on such plants to have a lower net reproduction rate (*R*_0_) and lower population density. This outcome may be due to a reduction in the aphid ingestion of both xylem and phloem, with the ingested phloem quantity setting the level of nutritional resources available to the aphids and the reduced xylem feeding interfering with the cotton aphids’ ability to ingest more phloem. In addition, higher levels of tannins in the drought-stressed cotton plants and lower levels of detoxifying enzymes in *A. gossypii* may also partially account for the poor development of *A. gossypii* on the drought-stressed plants. Overall, the changing growth and physiology of cotton plants under drought stress in turn have bottom-up effects on the population abundances of cotton aphids, but how physiological factors affect aphid performance needs further study. Furthermore, in future efforts to breed new drought-resistant cotton cultivars, such plant secondary defenses in phloem sap should also be considered.

## 4. Materials and Methods

### 4.1. Host Plants and Drought Treatments

*Gossypium hirsutum* cv. Zhongmian 49 used in this experiment was provided by the Institute of Cotton Research, Chinese Academy of Agricultural Science (Anyang, Henan Province). Seeds were sown in plastic pots (top diameter, 120 mm; bottom diameter, 90 mm; height, 100 mm) containing peat, vermiculite, and field soil (volume ratio: 6:1:1) in a greenhouse at 28–30 °C, 50 ± 5% RH, and 16:8 (L/D) h photoperiod. Plants were grown in these pots until the cotyledons were fully unfolded, and then plants with consistent growth were selected and moved into black plastic boxes (diameter: 2.5 cm, height: 3.5 cm), each containing 1 L half-strength modified Hoagland nutrient solution. The nutrient solution was replaced with fresh solution weekly. The Hoagland nutrient solution contained Ca(NO_3_)_2_·4 H_2_O (5 mmol L^−1^), KH_2_PO_4_ (1 mmol/L^−1^), K_2_SO_4_ (1 mmol L^−1^), MgSO_4_·7 H_2_O (2 mmol L^−1^), H_2_BO_3_ (0.045 mmol L^−1^), ZnSO_4_·7 H_2_O (8 × 10^−3^ mmol L^−1^), CuSO_4_·5 H_2_O (3 × 10^−3^ mmol L^−1^), MnSO_4_·4 H_2_O (6.7 × 10^−3^ mmol L^−1^), H_2_MoO_4_·4 H_2_O (5 × 10^−3^ mmol L^−1^), EDTA-Na2 (0.02 mmol L^−1^), and FeSO_4_·7 H_2_O (0.02 mmol L^−1^). Every growing box held 1 plant and was ventilated with an oxygen pump for 3–4 h daily. At the four-leaf stage, seedlings showing good growth were selected for use in drought-stress treatments. For our treatments, we chose 3 polyethylene glycol-6000 (PEG-6000) concentrations to simulate drought stress: 0 (the control), 1, and 3%. The 1% and 3% PEG-6000 levels represent moderate and severe drought stress to cotton plants, respectively. All plants in the experiment after imposition of treatments were held together in a greenhouse at 26 °C, 50 ± 5% RH, and 16: 8 (L/D) h photoperiod. After 28 d, cotton plants without aphid infestations were used to assay the relative water content, above-ground dry mass, and chemical composition (soluble sugar, soluble protein, N, and tannin) of the plants. 

### 4.2. Aphids

Cotton aphids, *A. gossypii*, were collected from cotton fields at the Korla Experimental Station of the Chinese Academy of Agricultural Sciences (Korla, Xinjiang Province) on 21 June 2019. The cotton in sampled field had been maintained without any pesticide applications before aphid collection. The aphid population comes from 1 parthenogenetic female aphid that was reared on 4- to 5-leaf stage potted cotton plants in a climate-controlled growth chamber with 26 ± 1 °C, 50 ± 5% RH, and 16: 8 (L/D) h photoperiod at the Langfang Experimental Station, Chinese Academy of Agriculture Science (Langfang, Hebei Province). 

### 4.3. Relative Water Content of Leaves and Above-Ground Dry Mass of Plants

All true leaves were sampled to analyze the relative water content (RWC) according to its formula RWC (%) = (fresh weight − dry weight) × 100/(turgor weight − dry weight) [55]. Sampled leaves from each plant were rinsed with distilled water for 1 s to clean the dust from the blades, then blotted with filter paper to remove water and weighed to obtain the fresh weight. The leaves were then rehydrated in distilled water for 24 h at 15 °C in darkness and weighed again to measure their mass at full turgor. Leaves were then dried at 105 °C for 30 min followed by 80 °C until reaching a constant dry weight. Each treatment had 10 replicates, 1 plant being a replicate.

The plant stems were cut at the soil level, and then the whole plant (stems and leaves) was weighed. Harvested plant tissues were then heated at 105 °C for 30 min and then dried at 80 °C, reaching a constant weight to obtain the above-ground dry weight. Each treatment had 10 replicates, with each replicate consisting of all above group parts from 1 plant.

### 4.4. Soluble Sugar, Soluble Protein, N, and Tannin Determination

Two uppermost fully expanded leaves of one plant were collected to characterize plant physiology. Three samples with six leaves were randomly chosen per treatment. Leaf samples were ground with liquid nitrogen using a mortar and pestle to measure soluble sugar, soluble protein, and N content. The anthrone colorimetric method was used to assay the soluble sugar concentration [56]. The Coomassie Brilliant Blue G-250 method (Bradford method) was used to analyze the soluble protein content [57] using the Reagent Kits (Kangwei Century Biotechnology Co., Ltd., Beijing, China). Kjeltec N method (Haineng Automated Kjeltec^TM^ instruments, Model K9860, Jinan, China) was used to determine the N content. The samples were freeze-dried at −80 °C for 24 h in the freeze dryer (VirTis genesis G25EL3, New York, NY, USA) and then ground to a fine powder (0.125 mm sieve) to determine the tannin content according to the directions of Reagent Kits (Solaibo Technology Co., Ltd., Beijing, China).

### 4.5. Effects of Drought Treatments on Aphid Life History, Population Abundance, and Changes in Metabolic Enzyme Levels

To test the effects of drought stress on cotton aphids at the individual level, three one-day-old apterous adult aphids were placed on the third true cotton leaves up from bottom that had been treated with PEG-6000 for seven days at one of three levels. Leaves were then covered with a 0.125 mm diameter mesh bag to prevent escape of aphids. After 12 h, the adult aphids were removed, and 1 first instar nymph was retained to measure the responses of individual cotton aphids to drought-stressed food plants. A total of 52, 35, and 35 newly emergency nymphs at the 3 levels of drought stress (0%, 1%, and 3% PEG-6000) were successfully used to analyze the aphids’ life history characteristics. Each test plant was fed on by only one nymph. The survival rate and developmental times of the test aphids were recorded daily. When the aphids molted to adults, their individual progeny were recorded daily and removed until all adults died.

To determine the effects of drought stress on cotton aphids at the population level, ten one-day-old apterous adult aphids were transferred onto the youngest fully expanded leaf on a test plant, which had been treated with one of three different levels of PEG-6000 stress for seven days. The plants with aphids were then housed separately in cages (28 L × 33 W × 53 cm H). Each cage held one plant. After 12 h, the adult aphids were removed, and five newly emerged nymphs were retained. The numbers of nymphs and adults on each plant were recorded on days 5, 10, 15, 20, 25, 30, 35, and 40. For each drought treatment level, there were 15 plants, with each plant being treated as 1 replicate.

At the end of the above experiment, the adult aphids only were brushed from each cotton plant and collected. Randomly, 30 adults from each plant were selected and analyzed for levels of 3 enzymes (glutathione-S-transferase, GST; carboxylesterase, CarE; mixed-functional oxidase, MFO). The activity levels of GST and CarE were measured using the micro glutathione-S-transferase and micro carboxylesterase Reagent Kits (Solaibo Technology Co., Ltd., Beijing, China), respectively. The activity of MFO was measured using the insect mixed-function oxidase ELISA Reagent Kit (Jianglai Biotechnology Co., Ltd., Shanghai, China). The aphids selected for measurement of enzyme levels were a mixture drawn from 15 plants. Each drought-stress treatment level was tested with 3 replicates, each with 30 aphids per replicate.

### 4.6. Aphid Feeding Behaviors

The feeding behaviors of adult aphids on cotton were recorded using the electrical penetration graph technique (EPG) on a Giga-8 direct current electrical penetration graph amplifier system with a 1 Giga Ω input resistance and an input bias current < 1 pA in a Faraday cage (manufactured by Wageningen University, Wageningen, the Netherlands) [50] as described by Liu et al. [24]. For feeding, tested adult aphids were placed on cotton plants in the 4-leaf stage that had been treated with simulated drought stress for 14 days. An eight-channel amplifier simultaneously recorded eight individual aphids on separate plants. Aphids were gently placed on the lower surface of a single cotton leaf (the third true leaf) and allowed to feed for 8 h. Feeding behaviors of aphids were recorded on plants subject to 3 drought-stress treatments simultaneously, and each treatment was randomly assigned to 2 or 3 of the 8 available channels. Waveform patterns were defined according to previously described categories [50] as follows: (1) non-penetration (NP); (2) initiation of stylet penetration of leaf tissue (which correlates with the intercellular apoplastic stylet pathway located in the epidermis/mesophyll cell layers in which the aphid shows a cyclic activity of mechanical stylet penetration and secretion saliva) (C); (3) short bouts of intracellular penetration, which leads to a signal potential drop (Pd); (4) secretion of saliva into phloem sieve elements at the beginning of the phloem phase (E1); (5) phloem ingestion (E2); (6) ingestion of xylem sap (G); and (7) time lost to mechanical difficulty in stylet penetration (F). Here, we selected the total durations of 6 behaviors, C, Pd, E1, E2, G, and F, to analyze the feeding responses of cotton aphids to drought-stressed cotton plants. EPG observations were made for 15 adult aphids for each of the 3 drought-level treatments.

### 4.7. Data Analysis

We used one-way ANOVA with SPSS software (SPSS 21.0, IBM, Armonk, NY, USA) to analyze the relative water content, above-ground plant biomass, physiology composition (soluble sugar, soluble protein, N content, and tannin content) of plants, aphid population abundance, and data on feeding behaviors of aphids feeding on plants. Tukey’s multiple range tests were used to determine differences among different drought treatments at *p* < 0.05. All life history data, such as developmental time, fecundity, longevity, age-stage specific survival rates *(s_xj_*) (*x* = age, *j* = stage), the age-specific survival rate (*l_x_*), the age-specific fecundity (*m_x_*), age-specific maternity (*l_x_m_x_*), and population parameters (the intrinsic of increase, *r*; the net reproductive rate, *R*_0_; the finite rate of increase, *λ*; the mean generation time, *T*), were analyzed using age–stage, two-sex life table methods with the computer program TWOSEX-MSChart [58]. The definitions and formulae for calculation of these parameters are in Chi and Liu [59] and Chi [60]. The standard errors of developmental time, fecundity, longevity, adult pre-reproductive period (APOP), and population parameters were analyzed using bootstrap approach with 100,000 resampling in computer program TWOSEX-MSChart [61,62]. Differences between different drought treatments were analyzed using a paired bootstrap test at 5% significance level. All graphs were made by GraphPad Prism 8.0 (GraphPad Software, La Jolla, CA, USA).

## Figures and Tables

**Table 1:** The effects of PEG-6000 stress on the developmental time and fecundity of *Aphis gossypii*. APOP is the pre-reproductive period based on the aphid adult stage. The numbers in parentheses are the numbers of aphid individuals at that specific PEG-6000 concentration. Different small letters indicate statistical differences in the same row between treatments by the paired bootstrap test in computer program TWOSEX-MSChart (*p* < 0.05).

Values	PEG-6000 Concentration		
	0% (52)	1% (35)	3% (35)
Nymph (d)	5.49 ± 0.16 b	5.67 ± 0.15 b	6.37 ± 0.17 a
Adult longevity (d)	33.60 ± 0.64 a	27.97 ± 1.19 b	26.97 ± 0.63 b
APOP (d)	0.43 ± 0.12 a	0.13 ± 0.08 b	0.13 ± 0.06 b
Fecundity (nymphs per adult female)	52.66 ± 2.07 a	34.40 ± 2.67 b	25.90 ± 1.45 c

**Table 2:** Population parameters of *Aphis gossypii* on cotton plants under different PEG-6000 concentrations. The numbers in parentheses are the numbers of aphid individuals at that specific PEG-6000 concentration. Different letters indicate statistical differences in the same row between treatments by the paired bootstrap test in the computer program TWOSEX-MSChart (*p* < 0.05).

Population Parameters	PEG-6000 Concentration		
	0% (52)	1% (35)	3% (35)
Intrinsic rate of increase (*r*) (d^−1^)	0.28 ± 0.01 a	0.27 ± 0.01 a	0.24 ± 0.01 b
Finite rate (*λ*) (d^−1^)	1.33 ± 0.01 a	1.32 ± 0.01 a	1.27 ± 0.01 b
Net reproduction rate (*R*_0_) (offspring)	47.60 ± 2.83 a	29.49 ± 3.03 b	22.20 ± 1.96 c
Mean generation time (*T*) (d)	13.66 ± 0.38 a	12.3 ± 0.33 b	13.02 ± 0.34 ab

## Data Availability

Not applicable.
